# Integrated physiological and molecular insights into photosynthetic responses of maize following relay-cropping of tobacco

**DOI:** 10.3389/fpls.2026.1787851

**Published:** 2026-05-07

**Authors:** Ruijia Ma, Bingjie Dai, Changsi Li, Shibo Wu, Zhixin Li, Rui Li, Fuzhao Nian, Leifeng Zhao, Yating Liu, Yanfen Xie, JiaBin Dong, Xiaolin Liao, Xiaopeng Deng, Di Liu

**Affiliations:** 1 College of Tobacco Science, Yunnan Agricultural University, Kunming, China; 2 College of Landscape and Horticulture, Yunnan Agricultural University, Kunming, China; 3 Hongyun Honghe Tobacco (Group) Co., Ltd. Qujing Cigarette Factory, Qujing, Yunnan, China; 4 Yunnan Tobacco Company, Qujing, Yunnan, China; 5 Zhongke Biotechnology (Yunnan) Co., Ltd., Kunming, Yunnan, China; 6 College of Food Science and Technology, Yunnan Agricultural University, Kunming, China; 7 Yunnan Academy of Tobacco Agricultural Sciences, Kunming, China

**Keywords:** C3, C4, maize, maize transcriptome, photosynthesis, species interaction, symbiotic period, tobacco

## Abstract

**Introduction:**

In the relay cropping system where maize growth overlaps with the tobacco harvesting period, clarifying light competition during the co-growth stage and its effects on maize photosynthesis is essential for optimizing cropping spatial configuration. This study aimed to reveal the physiological and molecular mechanisms underlying the photosynthetic response of silage maize (Huidan No. 4) to relay intercropping with flue-cured tobacco (K326).

**Methods:**

A field experiment was established with two treatments: maize relay-cropped after tobacco harvest and maize monoculture. At 15, 25, and 35 days after the formation of the intercropping competitive system, photosynthetic indices, chlorophyll content, and the activities of photosynthesis-related enzymes were determined in maize functional leaves (the third leaf from the top). Transcriptome sequencing was also performed to elucidate the molecular mechanism by which tobacco shading affects maize photosynthesis.

**Results:**

The solar radiation intercepted by relay-cropped maize was significantly lower than that of monoculture maize. Correspondingly, relay-cropped maize exhibited declined photosynthetic performance, with significant reductions in photosynthetic parameters, chlorophyll content, and activities of photosynthesis-related enzymes. Transcriptome analysis of maize functional leaves during the co-growth period identified a total of 3200 differentially expressed genes. KEGG pathway enrichment analysis showed that these differentially expressed genes were significantly enriched in photosynthesis-related pathways. Key genes involved in C3 and C4 photosynthetic pathways, including *PPC*, *PPDK*, *RBCL*, and *PRK*, were significantly downregulated in relay-cropped maize. Analysis of maize photosynthetic indices across tobacco–maize co-growth durations of 0–35 days indicated that the suitable symbiotic period was 0–25 days.

**Discussion:**

In the tobacco–maize relay intercropping system, the combined effects of severe shading and shading duration downregulate photosynthesis-related genes, inhibit the activities of key carbon fixation enzymes in maize, reduce carbon dioxide fixation capacity, and restrict the accumulation of organic matter in maize plants. This study systematically illustrates the regulatory mechanism of tobacco shading on maize photosynthetic characteristics at both physiological and molecular levels, providing a theoretical basis for optimizing the spatiotemporal layout of planting systems combining grain and economic crops.

## 1 Introduction

Tobacco (*Nicotiana tabacum*) is an annual herbaceous species in the Solanaceae family (genus *Nicotiana*), characterized by high biomass, rapid growth, wide adaptability, ease of cultivation, and strong amenability to genetic manipulation. It is extensively cultivated in Yunnan and Guizhou provinces of southwest China. Due to its economic and medicinal value, tobacco serves as an important model organism and is widely used in the production of food, feed, pharmaceuticals, chemical raw materials, and pesticides. Maize (*Zea mays*) is an annual herbaceous species of the Poaceae (grass family) and a major food crop in China. As a C4 plant, maize requires higher light intensity than C3 crops (Archie and Portis, 1995). Relay cropping improves land-use efficiency through spatial and temporal complementarity, shortens the overall growth period, and promotes balanced nutrient uptake. Moreover, root exudates from different species can enhance water-use efficiency and increase soil water storage in the root zone (Zhang et al., 2012) Flue-cured tobacco relay cropping with food crops such as soybean and maize can mitigate soil degradation caused by continuous tobacco monoculture, promote soil recovery, and increase food production. However, under field conditions, maize grown in relay systems with flue-cured tobacco often shows reduced growth during the tobacco harvesting period (Tao, 2023), primarily due to shading by large tobacco leaves, which intensifies competition for light (Huang et al., 2024). Despite this, the effects of low-light stress on maize growth and the underlying photosynthetic response mechanisms in such systems remain poorly understood. Transcriptome analysis enables the rapid identification of key regulatory genes associated with agronomic traits and reveals molecular adaptations to stress, facilitating the discovery of stress-responsive genes (Luo et al., 2025).

The C3 photosynthetic pathway, also known as the Calvin cycle, involves the fixation of atmospheric CO_2_ by ribulose-1,5-bisphosphate carboxylase/oxygenase (Rubisco). The initial product is a three-carbon compound, 3-phosphoglycerate (PGA), hence the name C3 pathway. Plants relying on this pathway are classified as C3 plants (Zeng, 2023). The key enzyme Rubisco catalyzes both carboxylation and oxygenation of RuBP (Archie and Portis, 1995; Bernacchi et al., 2001; Parry et al., 2002), thereby reducing carbon assimilation efficiency and constraining photosynthesis. To overcome reduced CO_2_ availability under stress conditions, such as stomatal closure at high temperatures, and to limit losses from oxygenation, plants evolved the more efficient C_4_ pathway (Daoura-Gaoh-Goudia, 2017). In C_4_ plants, initial CO_2_ fixation is catalyzed by phosphoenolpyruvate (PEP) carboxylase, which has a high CO_2_ affinity and maintains strong carboxylation activity even under low ambient CO_2_ conditions (Su, 2008; Su et al., 2011). This reaction produces oxaloacetate, a four-carbon compound (Li et al., 1999), which is transported to adjacent bundle sheath cells. There, CO_2_ is released and enters the C_3_ cycle. This mechanism functions as a CO_2_-concentrating system, creating a high-CO_2_ microenvironment around Rubisco and enhancing carboxylation efficiency, thereby supporting efficient completion of the photosynthetic carbon cycle. The C4 pathway is classified into three subtypes: PEPCK (Jiang et al., 2011; Sun et al., 2024), NAD-ME (Sadr et al., 2023; Vargas et al., 2024), and NADP-ME (Ward and Woolhouse, 1986). Maize belongs to the NADP-ME subtype (Detarsio et al., 2008; Hasan et al., 2005; Rothermel and Nelson, 1989). Unlike C_3_ species, C_4_ plants integrate both C_3_ and C_4_ cycles and typically require higher light intensity and temperature. Photosynthesis directly determines plant growth and yield. Weak light stress impairs photosynthesis and reduces growth and yield in both C_3_ crops, such as potato (Qin et al., 2014; Zhou et al., 2024) (e.g., potatoes) and C4 crops (e.g., maize). Therefore, understanding carbon fixation in C_4_ plants under weak light stress induced by relay cropping is critical for optimizing cropping schedules. This study examines the photosynthetic response of the silage maize cultivar Huidan No. 4 during relay cropping with flue-cured tobacco under field conditions, from seedling emergence to tasseling.

## 2 Materials and methods

### 2.1 Experimental materials and treatments

**Figure 1:**
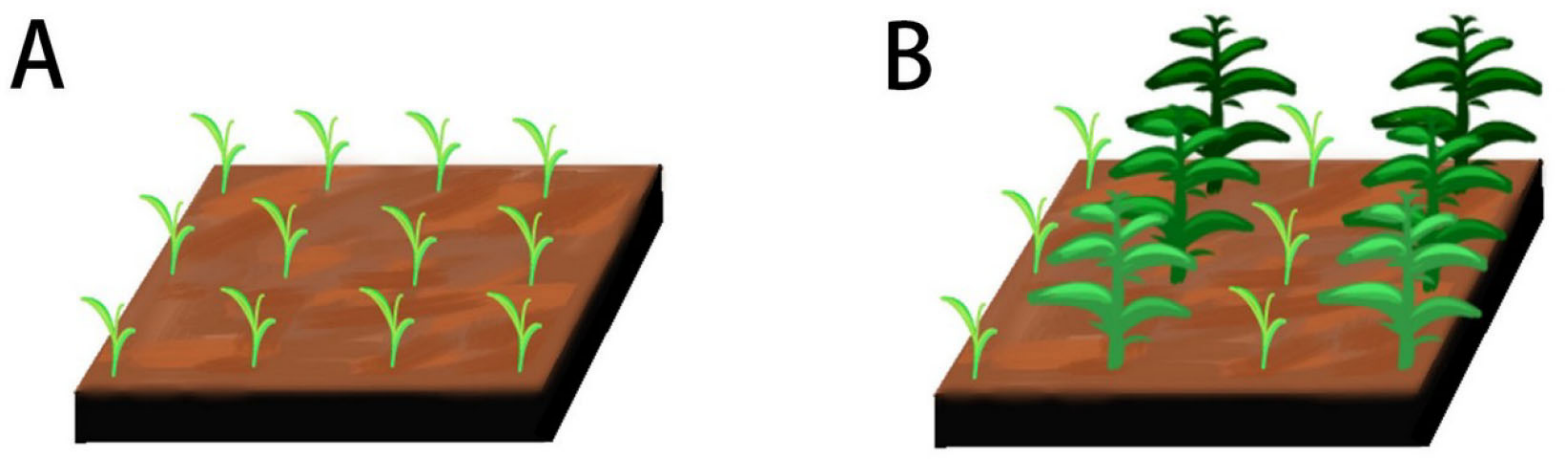
Model diagram of field test. **(A)** Model map of monoculture maize in field; **(B)** Model map of maize relay cropping of flue-cured tobacco field.

The field experiment was conducted in Xiaoyangzhai Village, Zhushan Township, Yiliang County, Kunming City, Yunnan Province (24°30′36” N, 102°58′22” E) at an altitude of 1,600 m. The site has an average annual temperature of 15.91 °C, annual rainfall of 850 mm (mainly from May to October), and an average annual sunshine duration of 2,177.3 h. A single-factor field experiment included two treatments: (1) IM: relay intercropping of silage maize Huidan No. 4 (Yunnan Guangda Seed Industry Co., Ltd.) with flue-cured tobacco K326 (Yuxi Zhongyan Seed Co., Ltd.); and (2) MM: maize monoculture. In the relay system, maize was sown on both sides of tobacco rows after harvesting the lower tobacco leaves (**Figure 1A**). In the monoculture treatment, maize was sown simultaneously in open fields without tobacco (**Figure 1B**). Under the condition of uniform soil fertility, two treatments were each replicated with three adjacent plots, and each plot occupied an area of 0.044 ha. Flue-cured tobacco was planted at 120 cm row spacing and 50 cm plant spacing. At transplanting, 20 kg of compound fertilizer (N:P_2_O_5_:K_2_O = 12:6:24) was applied as a basal dose. At the budding stage, 300 kg/ha of the same fertilizer plus 10 kg/ha K_2_SO_4_ were applied. During vigorous growth, KH_2_PO_4_ was applied as a foliar spray at 15 kg/ha.

For relay cropping, tobacco seedlings were raised for 45 days and transplanted in late April 2023, and maize was sown on August 2, 2023. During the coexistence period, tobacco leaves were harvested at 15, 25, and 35 days after maize sowing, with the final harvest marking the end of this stage. Relay cropping maize was sown immediately after harvesting the lower tobacco leaves, on both sides of each tobacco row. In the monoculture plots, any remaining tobacco plants were removed. Both relay-cropped and monoculture maize were planted in a two-row arrangement with 40–45 cm row spacing, 25–30 cm plant spacing, and a sowing depth of 3–5 cm, resulting in a density of 55,500–66,000 plants per hectare. Field management followed local practices. At the end of the symbiotic stage, urea was applied at 600 kg/ha as the first topdressing, followed by diammonium phosphate at 600 kg/ha at 50 days after sowing. Plant samples were collected at 15, 25, and 35 days during the coexistence stage for subsequent measurements.

### 2.2 Plant sample collection and measurement

The experiment consisted of two treatments, with three plots repetitions per treatment. For sampling, three random samples were randomly selected from each plot, and then the three samples from the three plots are combined to form a composite sample. This process is repeated 3 times to generate 3 composite samples. Light intensity was measured at 0, 15, 25, and 35 days after maize sowing using a five-point sampling method. Measurements were taken on functional leaves (third leaf from the top) of maize grown under relay cropping and monoculture using an SM206-PAR quantum sensor (Shenzhen Xinbaorui Instrument Co., Ltd.). Day 0 (the day before sowing) served as the baseline for early light conditions in the coexistence system. On days 15, 25, and 35, functional leaves (third fully expanded leaf from the top, which provides a more consistent and representative measure of photosynthetic performance) were collected from both treatments to assess key photosynthetic parameters, including net photosynthetic rate (Pn), intercellular CO_2_ concentration (Ci), transpiration rate (Tr), and stomatal conductance (Gs) (n = 3). For each sample, 5 g of leaf tissue was placed in cryovials, flash-frozen in liquid nitrogen, and stored at −80 °C for subsequent analysis of Rubisco, PEPC, and PPDK activities (Suzhou Genesis Biotechnology Co., Ltd.) and transcriptome sequencing (Guangzhou Genedenovo Technology Service Co., Ltd.).

Real-time fluorescence quantitative PCR (qRT-PCR) analysis was performed as follows. Total RNA was extracted from leaves using TRIzol. First-strand cDNA was synthesized from 1 μg of total RNA with a reverse transcription kit. The qRT-PCR assay was conducted on a ABI 7500 real-time fluorescence quantitative PCR system using the SYBR Green fluorescent dye method. Six representative differentially expressed genes (DEGs) were selected, and gene-specific primers were designed using Primer 5.0 software (**Supplementary Table 2**). The EF1α was used as an internal control, and the relative expression levels of target genes were calculated using the 2−ΔΔCt method. All reactions were performed in triplicate.

Maize yield was measured according to the Ministry of Agriculture and Rural Affairs of the People’s Republic of China” (Ministry of and Rural Affairs of the People's Republic of, 2019). Plants were cut 20 cm above ground, and fresh biomass per plot was weighed immediately to determine yield, which was then converted to kg ha^-^¹. Plots with more than 10% missing plants were excluded from the analysis.

### 2.3 Statistical analysis

Statistical analysis was performed using ANOVA in SPSS 22.0, with significance set at *P* < 0.05. Graphs were generated using GraphPad Prism 8.0.2.

## 3 Results and analysis

### 3.1 Impact of relay cropping maize on yield

According to the final statistical analysis (**Table 1**), after a 35-day symbiotic period, relay cropping maize yielded 34.6 t/ha compared with 38.9 t/ha in monoculture, representing a significant reduction of 4.3 t/ha (9.51%). These results indicate that 35 days of field coexistence with flue-cured tobacco significantly suppresses maize growth and reduces yield. .

**Table 1:** Aboveground biological yield of maize under two cropping systems.

Maize variety	Planting pattern	The aboveground biological yield of maize(t/ha)
Huidan No.4	relay cropping maize	34.6 ± 1.183
	monoculture maize	38.9 ± 0.929*

ns indicates no significant difference, * indicates a difference at a significance level of 0.05 (p < 0.05), ** indicates a difference at a significance level of 0.01 (p < 0.01), and *** indicates a difference at a significance level of 0.001 (p < 0.001).

### 3.2 Impact of relay cropping maize on light radiation intensity

**Figure 2:**
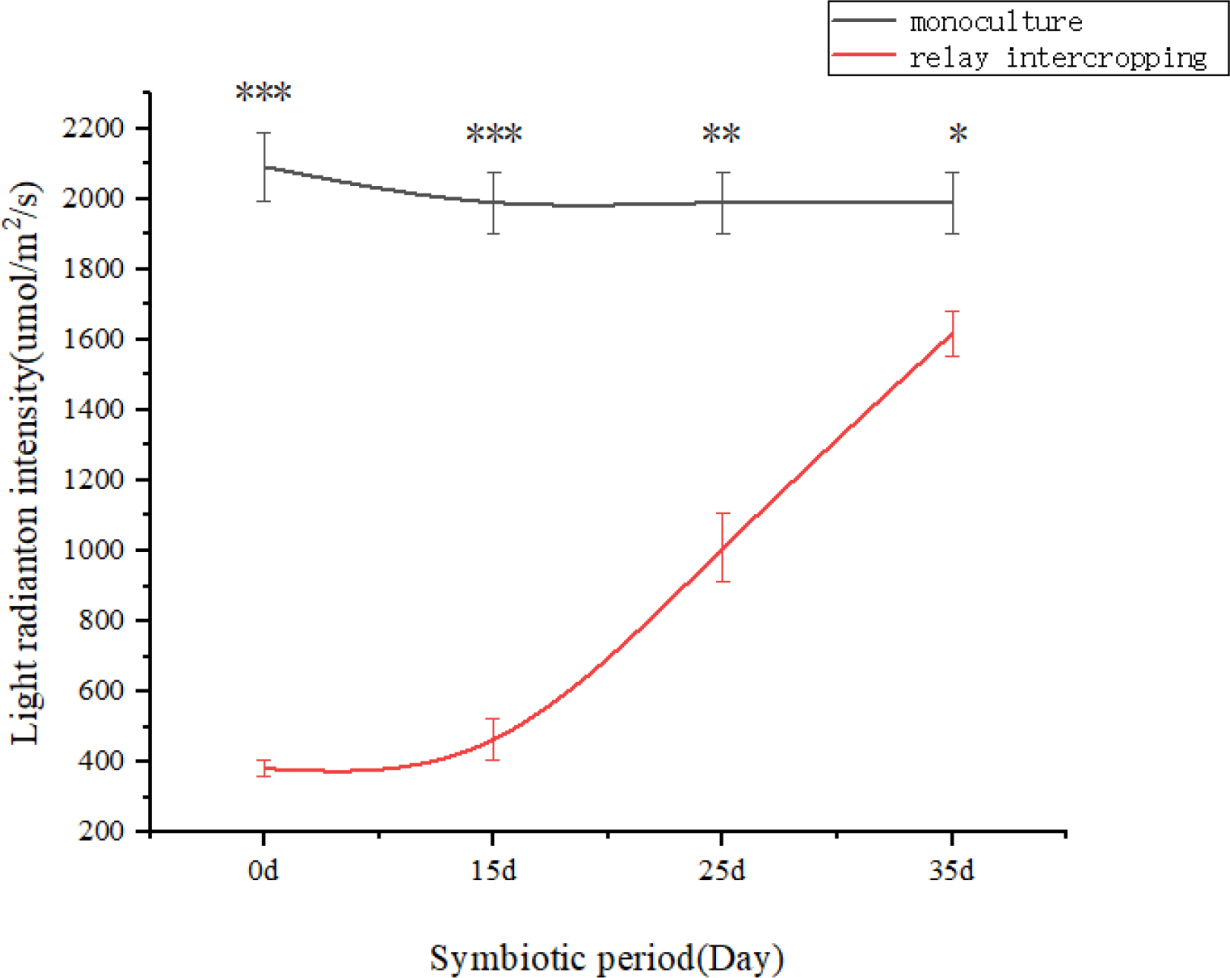
Light radiation intensity during different symbiotic periods of flue-cured tobacco and maize in the field.Note: ns indicates no significant difference, * indicates a difference at a significance level of 0.05 (p< 0.05), ** indicates a difference at a significance level of 0.01 (p < 0.01), and *** indicates a difference at a significance level of 0.001.

According to the field test data (**Figure 2**). On the day before maize sowing, when tobacco retained 12–14 leaves, light intensity at 2 cm above the ground under the tobacco canopy was significantly lower than in the open monoculture field, establishing the baseline light environment for relay cropping maize. During subsequent sampling at 15 (12–14 leaves), 25 (8–9 leaves), and 35 days (2–3 leaves), light intensity in relay cropping maize remained consistently and significantly lower than in monoculture, confirming sustained light limitation. Although partial recovery occurred between 15–25 and 25–35 days after sowing following harvest of middle and upper tobacco leaves, light availability remained significantly lower than under monoculture conditions. These findings demonstrate that tobacco canopy shading substantially reduced light intensity reaching maize. .

### 3.3 Effects of relay cropping with tobacco on maize photosynthetic parameters

**Figure 3:**
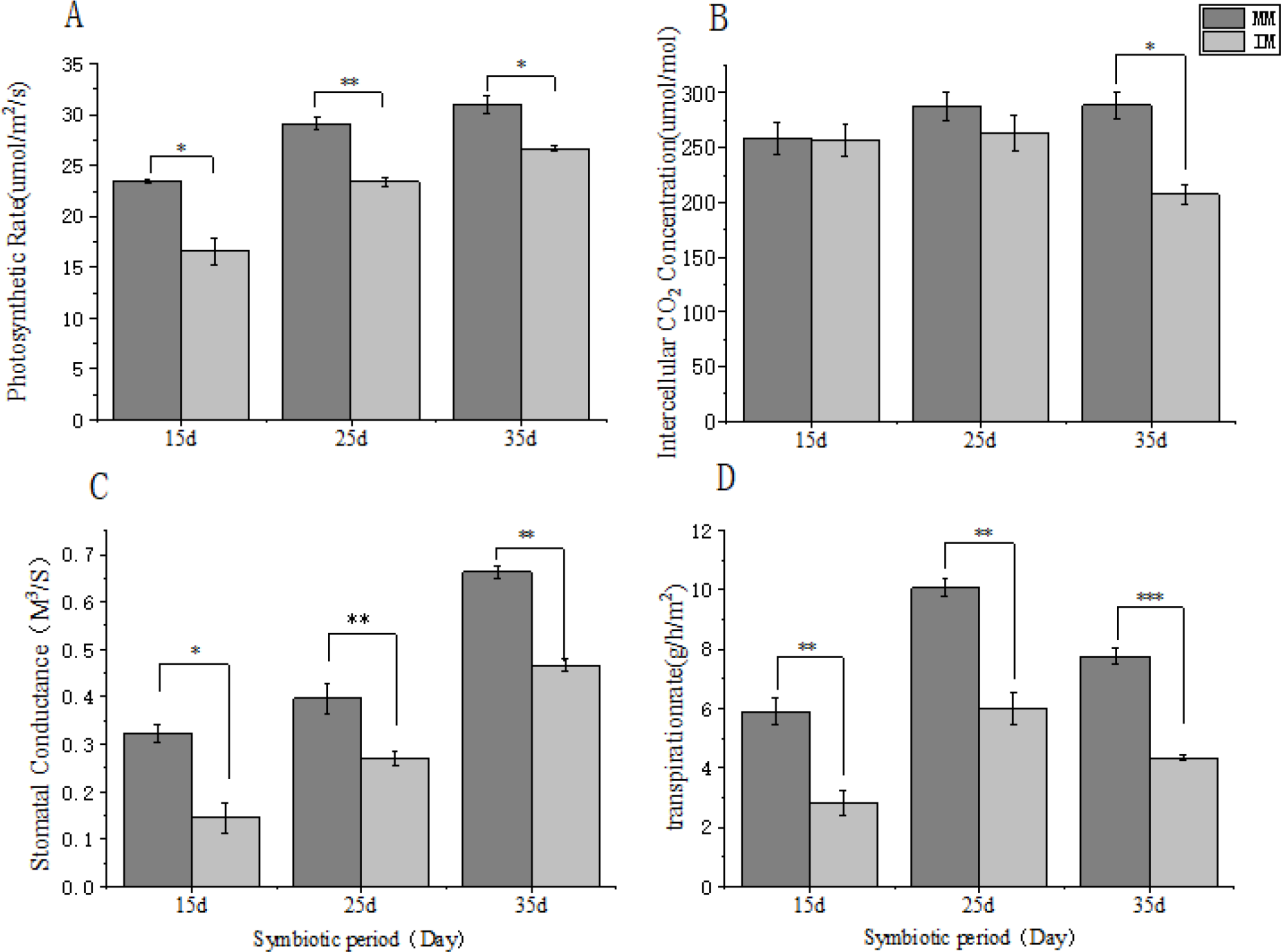
Effects of relay cropping of maize with flue-cured tobacco on photosynthetic parameters of maize at different symbiotic periods. **(A)** Net photosynthetic rate; **(B)**, intercellular CO_2_ concentration; **(C)** stomatal conductance; **(D)** transpiration rate. Data are presented as mean ± SD, standard deviation of three biological replicates and were analyzed using one-way ANOVA followed by Tukey’s test. Note: ns indicates no significant difference, * indicates a difference at a significance level of 0.05 (p< 0.05), ** indicates a difference at a significance level of 0.01 (p<0.01), and *** indicates a difference at a significance level of 0.001.

Photosynthetic performance was assessed using key parameters. As shown in **Figure 3**, relay cropping maize exhibited significantly lower net photosynthetic rate (Pn) and stomatal conductance (Gs) than monoculture maize at both 15 and 25 days after sowing. By 35 days, Pn, Gs, and intercellular CO_2_ concentration (Ci) remained significantly reduced. These results indicate that photosynthetic differences emerged as early as 15 days and that relay cropping maize experienced sustained shading stress from the seedling stage onward, with progressively increasing suppression over time.

### 3.4 Effects of relay cropping maize with tobacco on maize leaf enzyme activity

**Figure 4:**
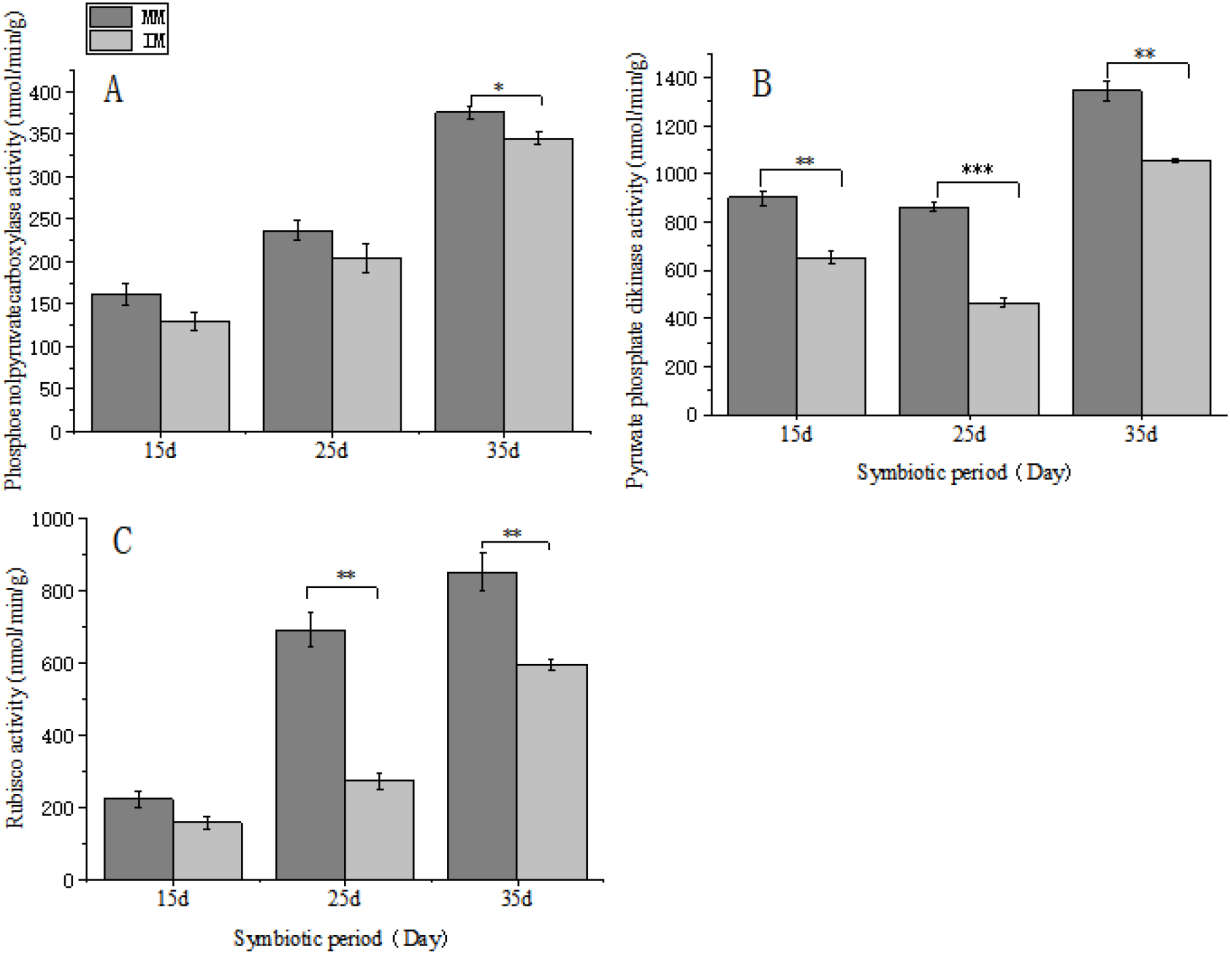
Effects of relay cropping of maize with flue-cured tobacco on enzyme activities of maize leaves at different symbiotic periods. **(A)** Phosphoenolpyruvate carboxylase activity; **(B)** pyruvate phosphate dikinase activity; **(C)** ribulose-1, 5-bisphosphate carboxylase activity. Data are presented as mean ± SD of three biological replicates and were analyzed using one-way ANOVA followed by Tukey’s test. ns indicates no significant difference, * indicates a difference at a significance level of 0.05 (p< 0.05), ** indicates a difference at a significance level of 0.01 (p<0.01), and *** indicates a difference at a significance level of 0.001.

This study also evaluated the activities of key enzymes in the C4 and C3 photosynthetic pathways, including pyruvate phosphate dikinase (PPDK), phosphoenolpyruvate carboxylase (PEPC), and ribulose-1,5-bisphosphate carboxylase/oxygenase (Rubisco). Compared with monoculture maize, PPDK activity was significantly lower at 15 days after sowing. At 25 days, both PEPC and Rubisco activities were markedly reduced, and by 35 days, PPDK and Rubisco activities again showed significant decreases (**Figure 4**). These results indicate that relay cropping with tobacco significantly inhibits key photosynthetic enzyme activities in maize.

### 3.5 Analysis of differentially expressed genes in monoculture maize and relay cropping maize

#### 3.5.1 Transcriptome sequencing and data quality assessment

**Figure 5:**
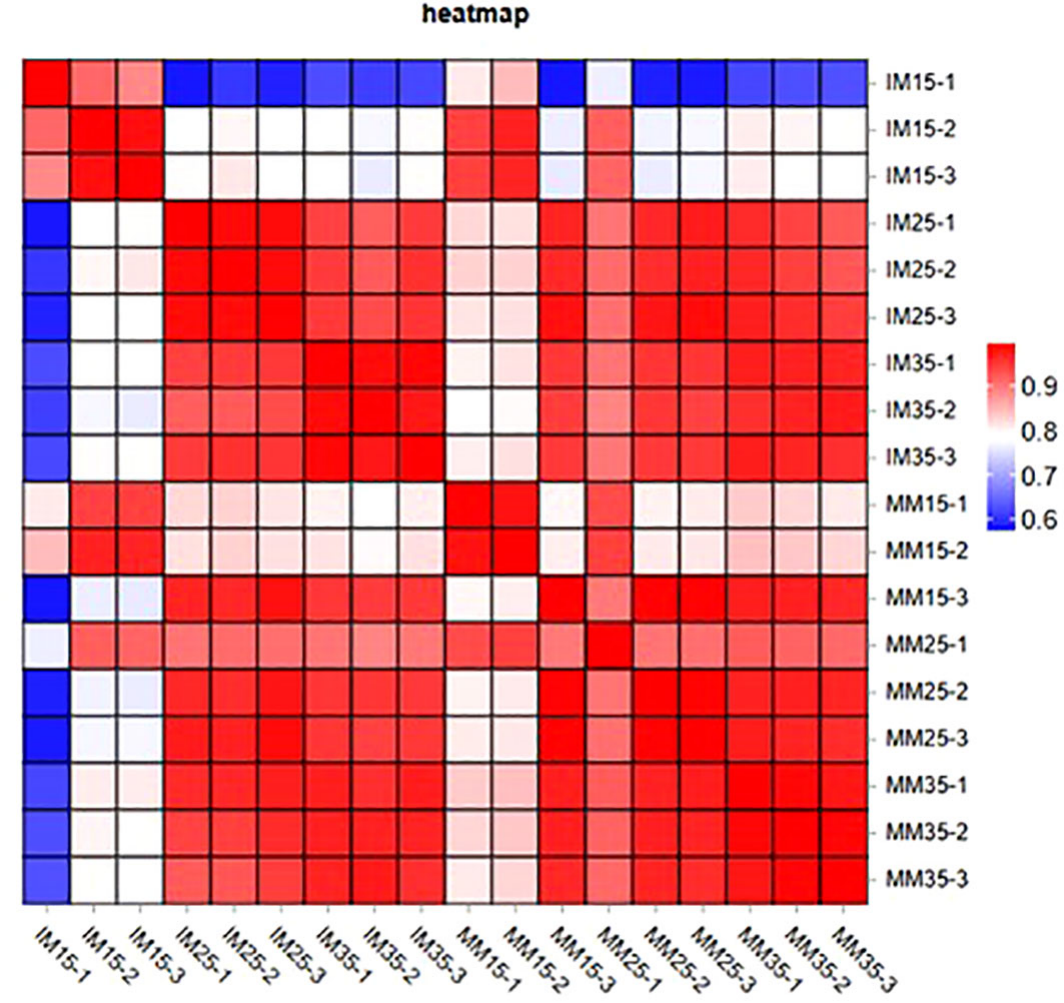
Pearson correlation coefficient of transcriptome dataset.

To elucidate the molecular mechanisms underlying maize photosynthetic responses under relay cropping with tobacco, RNA-based transcriptome sequencing was performed on maize leaf samples collected at different stages of co-growth with tobacco. Transcriptome data from 18 samples (three biological replicates per stage) showed high-quality coverage (**Supplementary Table 2**). After transcript quantification, 3,200 differentially expressed genes (DEGs) were identified. Biological replicates showed good reproducibility, with Pearson correlation coefficients (r) exceeding 0.58 across all groups (n = 3) (**Figure 5**), indicating reliable data for differential expression analysis (Nomura et al., 2025). Correlation coefficients ranged from 0.80 to 1.00 within groups and from 0.58 to 0.99 between groups, confirming strong intra-group consistency and acceptable inter-group comparability.

#### 3.5.2 Differential gene expression analysis

**Figure 6:**
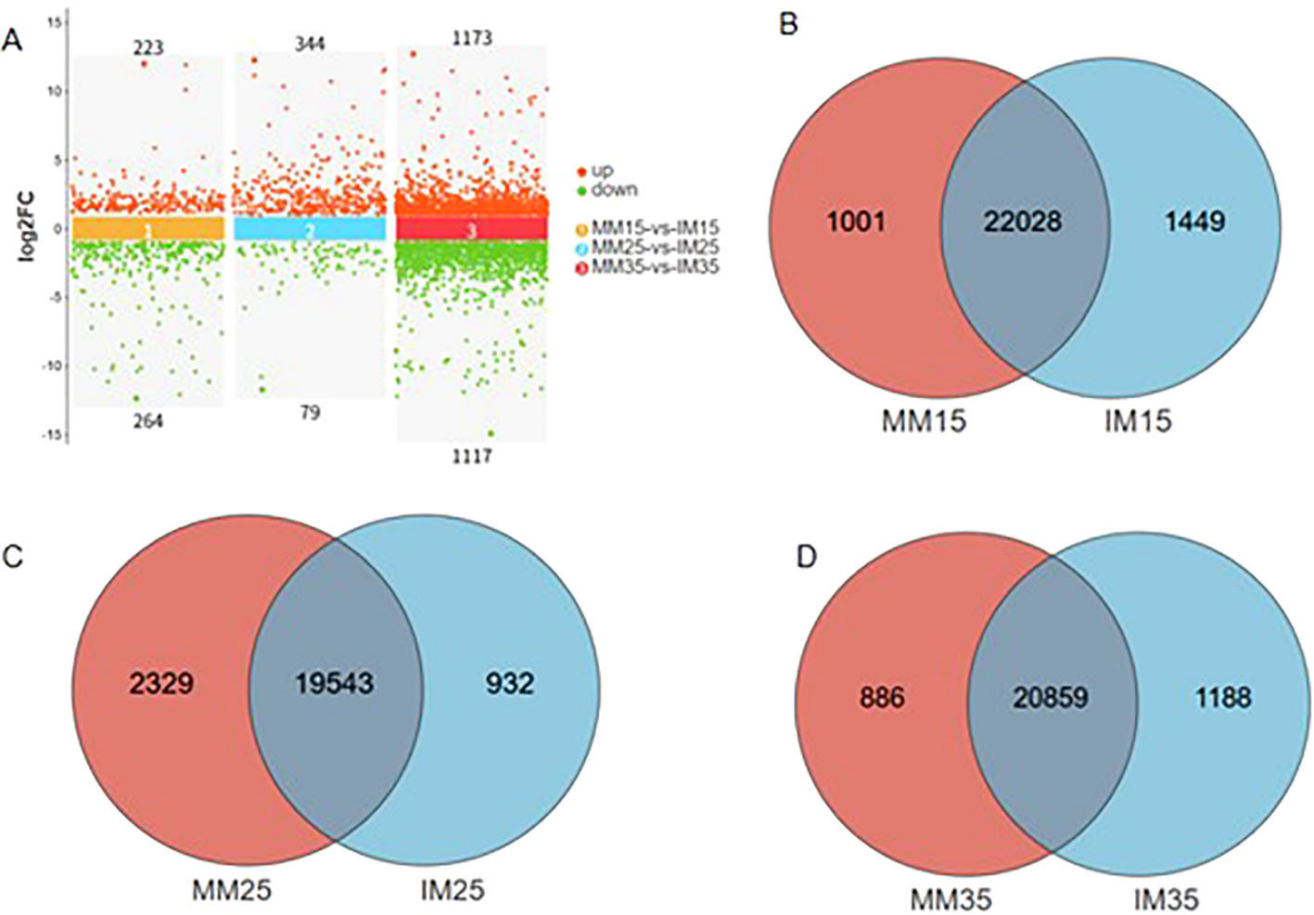
Distribution of differentially expressed genes at different symbiotic stages between tobacco and maize. **(A)** Venn diagram of MM15 vs. IM15 expressed genes; **(B)** Venn map of MM25 vs. IM25 expressed genes; **(C)** Venn diagram of MM35 vs. IM35 expressed genes; **(D)** Venn diagram of maize expressed genes across different treatment groups; F, number of differentially expressed genes in maize at different symbiotic stages.

To assess gene expression differences between relay cropping and monoculture maize, DEGs were identified at 15, 25, and 35 days after sowing using thresholds of |log2FC| ≥ 2 and FDR < 0.05 (**Figure 6**). At 15 days, 2,028 DEGs were detected, including 1,001 unique to monoculture and 1,449 unique to relay cropping. At 25 days, 19,543 DEGs were identified, with 2,329 and 932 unique to monoculture and relay cropping, respectively. At 35 days, 20,859 DEGs were observed, including 1,188 unique to monoculture and 886 unique to relay cropping. Pairwise comparisons between the two systems at each time point identified 487 significant DEGs at 15 days (223 upregulated, 264 downregulated), 423 at 25 days (344 upregulated, 79 downregulated), and 2,290 at 35 days (1,173 upregulated, 1,117 downregulated). The sharp increase in DEGs at 35 days indicates pronounced transcriptional reprogramming at the late intercropping stage.

#### 3.5.3 GO and KEGG enrichment analysis of maize

**Figure 7:**
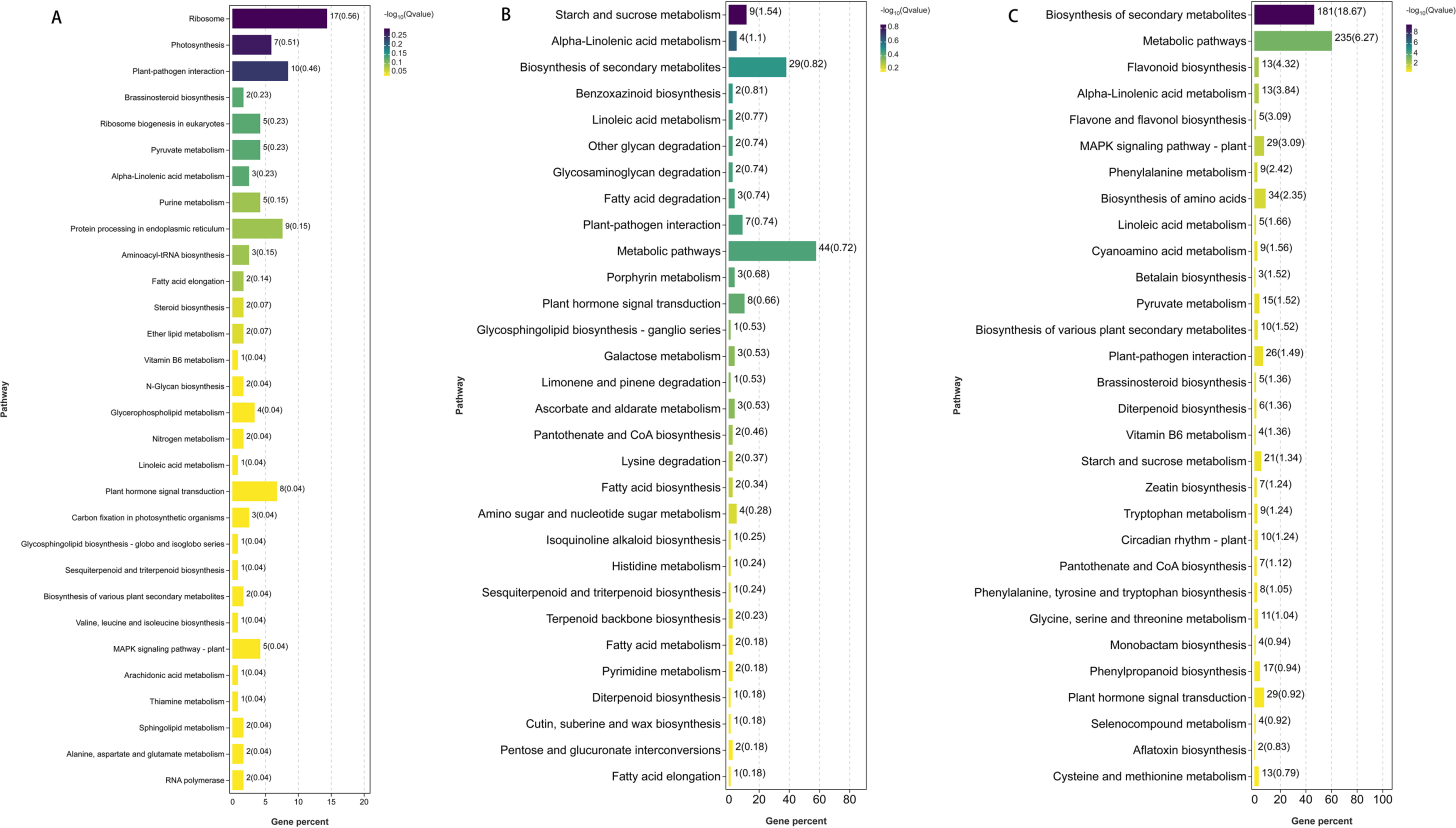
KEGG enrichment bar chart. The top 20 pathways with the smallest Q values are shown. The y-axis represents pathways, and the x-axis represents the enrichment factor (number of differentially expressed genes in a pathway divided by the total number of genes in that pathway). Point size indicates gene number, and red color intensity indicates smaller Q values. **(A)** shows MM15–IM15; **(B)** shows MM25–IM25; **(C)** shows MM35–IM35.

KEGG enrichment analysis of the top 20 significant DEGs at each stage (**Figure 7**) revealed stage-specific pathway shifts. At 15 days, enriched pathways included ribosomes, photosynthesis, and plant–pathogen interaction. At 25 days, enrichment shifted to starch and sucrose metabolism, biosynthesis of secondary metabolites, and phenylpropanoid biosynthesis. At 35 days, enriched pathways were primarily associated with secondary metabolism, general metabolic processes, and linoleic acid metabolism, all closely linked to photosynthetic function.

**Figure 8:**
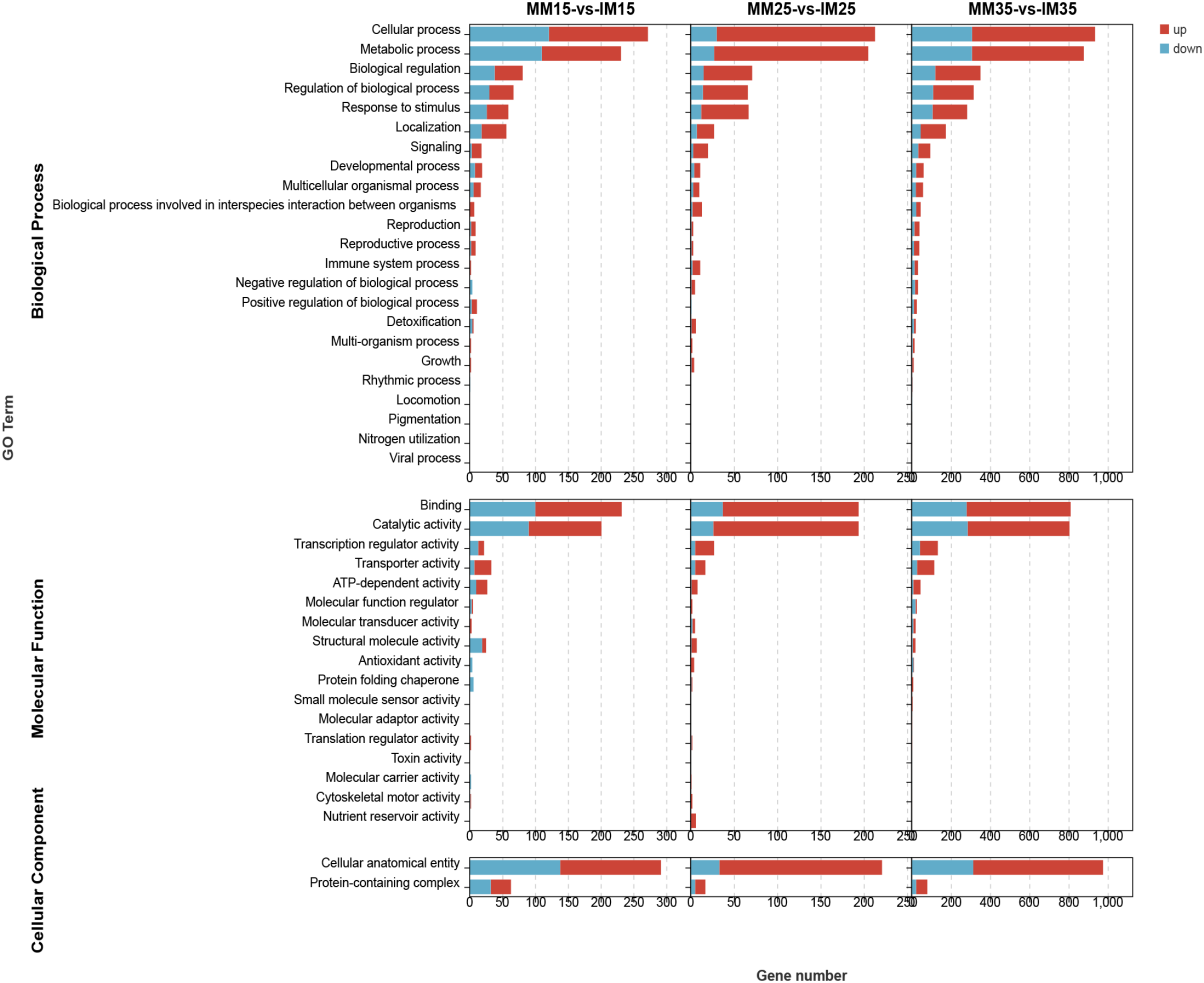
GO enrichment bar chart. The top 20 pathways with the smallest Q values are shown. The y-axis represents pathways, and the x-axis represents the enrichment factor (ratio of differentially expressed genes to total genes in the pathway). Point size indicates gene count, and color intensity (red) indicates lower Q values.

GO enrichment analysis (**Figure 8**) showed that DEGs in monoculture maize were classified into three categories: biological processes (BP), cellular components (CC), and molecular functions (MF). MF terms were mainly associated with protein-containing complexes and cellular anatomical entities, while CC was enriched for cellular anatomical structures. Overall, these annotations indicate that relay cropping–induced transcriptional changes are closely linked to metabolic and structural components of photosynthesis.

#### 3.5.4 Identification of C4 pathway marker genes in maize

**Figure 9:**
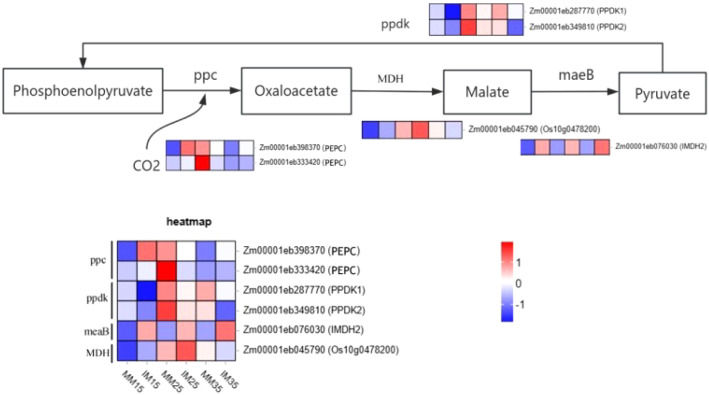
Gene expression patterns of maize NADP-ME (C_4_) pathway enzymes. *PPC*, phosphoenolpyruvate carboxylase; *MDH*, malate dehydrogenase; *PPDK/ppdk*, pyruvate phosphate dikinase; *meaB*, malate dehydrogenase.

To assess the photosynthetic response of relay cropping maize under low-light stress, we analyzed genes in the NADP-ME carbon production pathway and identified six DEGs (**Figure 9**): two phosphoenolpyruvate carboxylase genes (*PEPC*), two pyruvate phosphate dikinase genes (*PPDK1 and PPDK2*), and two malate dehydrogenase genes (*meaB* and *MDH*). The malate dehydrogenases represented two functional groups: *meaB* catalyzes the conversion of oxaloacetate to malate in the cytoplasm, whereas *MDH* converts malate to pyruvate in the chloroplast.

At 15 days after sowing, *MDH* and *PPDK* expression was markedly lower in relay cropping maize, whereas *meaB* and *PPC* were significantly upregulated. By 25 days, *PPDK* and *PPC* expression decreased, while *MDH* and *meaB* remained elevated. This pattern indicates increased transcription of *meaB* and reduced transcription of *PPDK* and *PEPC* during this period, consistent with previously observed declines in *PEPC* and *PPDK* enzyme activities, and suggests that photosynthetic acclimation was most pronounced at 25 days. At 35 days, redox-related genes (*meaB* and *PPC*) were upregulated, whereas *PPDK* and *MDH* were downregulated. Overall, carbon production–related genes, including *PPDK*, showed a consistent downregulation trend in relay cropping maize, similar to the initial CO_2_ receptor gene *PPC*, whereas *meaB* and *MDH* exhibited sustained upregulation.

#### 3.5.5 Identification of C3 pathway marker genes in maize

**Figure 10:**
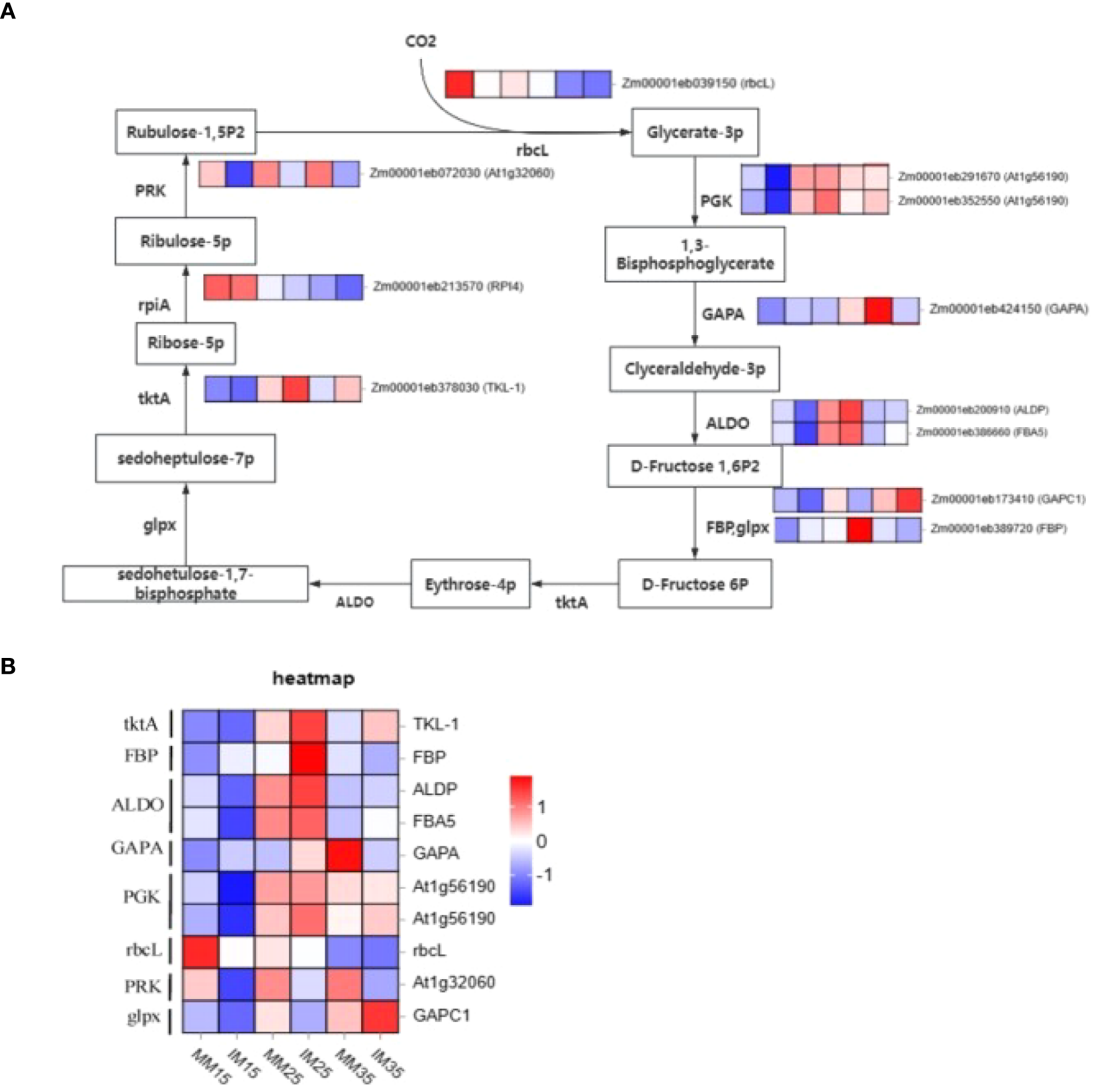
Heat map of differentially expressed genes associated with the C_3_ pathway in maize across symbiosis stages. **(A)** Expression patterns of enzyme-related genes in the C₃ pathway; **(B)** Heat map of differentially expressed genes related to the C₄ pathway. *TktA*, transketolase; *FBP*, fructose-1, 6-bisphosphatase; *ALDO*, fructose-bisphosphate aldolase; *GAPA*/gap, glyceraldehyde-3-phosphate dehydrogenase; *PGK*, phosphoglycerate kinase; *rbcL*, ribulose-1,5-bisphosphate carboxylase; *PRK*, phosphoribulokinase; *glpx*, fructose-1,6-bisphosphatase.

In this study, we identified 11 DEGs functionally linked to the Calvin cycle (**Figure 10**), a central pathway for photosynthetic carbon fixation and organic synthesis (Li et al., 2024). These included single genes encoding transketolase (*TKTA*), fructose-1,6-bisphosphatase (*FBP)*, glyceraldehyde-3-phosphate dehydrogenase *(GAPA)*, and phosphoribulokinase *(PRK)*, along with two genes each for fructose-bisphosphate aldolase *(ALDO)*, phosphoglycerate kinase *(PGK)*, ribulose-1,5-bisphosphate carboxylase/oxygenase *(rbcL)*, and ribose-5-phosphate isomerase *(rpiA).*

Expression analysis revealed stage-specific shifts in these Calvin cycle genes under relay cropping. At 15 days after maize sowing, *ALDO*, *GAP, PGK, rbcL*, and *PRK* were downregulated, whereas *FBP, GAPA*, and *rpiA* were upregulated. By 25 days, *tktA, FBP, ALDO*, and *PGK* were upregulated, while *GAPA, rbcL*, and *PRK* were downregulated. At 35 days*, tktA, FBP, ALDO, GAP*, and *PGK* were upregulated, whereas *GAPA, rbcL, PRK*, and *rpiA* were downregulated. Consistent with the C_4_ cycle, *PRK* and *rbcL* remained suppressed, paralleling trends observed in C_4_-cycle genes at 25 days under relay cropping, indicating reduced synthesis of the CO_2_ acceptor ribulose-1,5-bisphosphate and 3-phosphoglycerate (*PGA*). The significant decline in Rubisco activity in relay-cropped maize was consistent with lower *rbcL* expression, supporting the transcriptomic data.

#### 3.5.6 Validation of RNA-seq data by qRT-PCR

**Figure 11:**
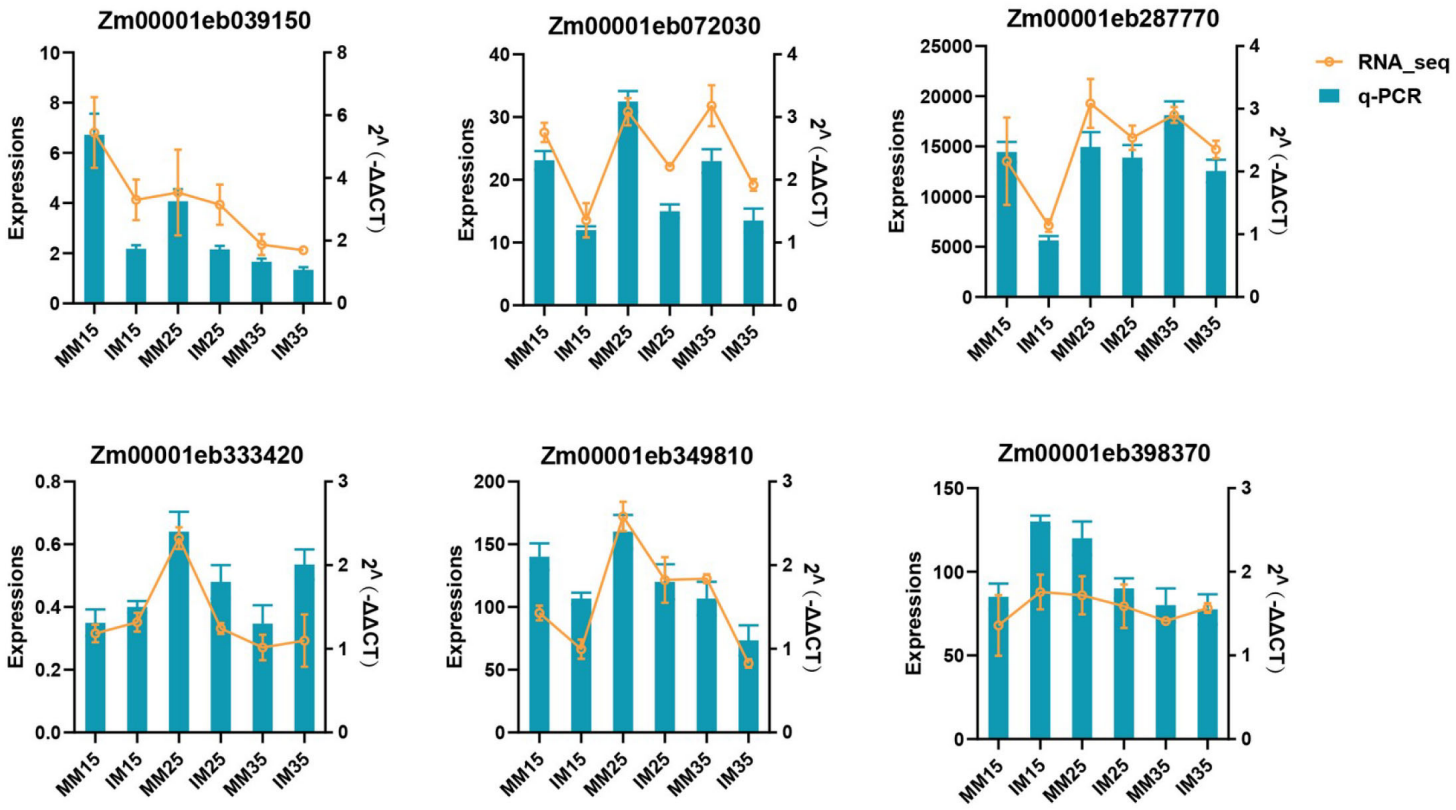
Comparing the detection results of qRT-PCR and RNA-seq using a dual-coordinate graph. The legend is displayed on the right side.

The results of qRT-PCR were compared with the changing trend of TPM values (**Figure 11**). It is worth noting that the qRT-PCR results were highly consistent with the expression patterns in the RNA-seq data, further verifying the accuracy of the RNA-seq data.

## 4 Discussion

### 4.1 Effects of relay cropping maize with tobacco on photosynthesis during harvesting period

Photosynthetic parameters are direct indicators of photosynthetic activity (Deng et al., 1984). In this study, relay-cropped maize was shaded by tobacco leaves, which reduced incident light. Wen et al (Wen et al., 2025) reported that low light decreases net photosynthesis, transpiration, and stomatal conductance in Moso bamboo, consistent with our results, where intercropped maize showed a significantly lower net photosynthetic rate than monoculture maize. Su et al (Su et al., 2011) also showed that elevated ambient CO_2_ induces stomatal closure and reduces transpiration, aligning with our findings. Because of its lower canopy position, intercropped maize was exposed to CO_2_ released from tobacco respiration and exhibited significantly lower transpiration than monoculture maize. Reduced light availability directly suppressed photosynthesis in relay-cropped maize. This suppression, together with higher within-canopy CO_2_ levels in the corn–alfalfa system, as reported previously (Buchmann and Ehleringer, 1998), led to decreased stomatal conductance and carbon-fixing enzyme activity, leading to CO_2_ accumulation in leaves. In contrast, photosynthesis in monoculture maize proceeded normally, resulting in no substantial difference in leaf CO_2_ concentration between systems. Additionally, C4 crops typically require less CO_2_ and assimilate it more efficiently, indicating that CO_2_ concentration may not be the primary limiting factor (L. Q. Huang and Guo, 2011). However, at 35 days, intercellular CO_2_ concentration was significantly lower in relay-cropped maize than in monoculture maize, indicating possible structural or physiological adaptations. These may include changes in photosystems (Ge et al., 2007), leaf anatomy under low light, or membrane damage (Lin, 1999), which require further investigation.

### 4.2 Effects of relay cropping maize with tobacco on photosynthetic enzymes in the harvest stage

Maize (Bellasio and Griffiths, 2014) uses the NADP-ME subtype of the C4 dicarboxylic acid cycle, in which CO_2_ is initially fixed via PEPC to form malate or aspartate. These intermediates subsequently enter the tricarboxylic acid cycle for energy metabolism. Compared with C3 species, C4 crops such as maize show higher photosynthetic efficiency and greater tolerance to abiotic stress, contributing to higher yield potential (Jafarpour and Nulit, 2011; Zheng et al., 2023). However, the specialized carbon assimilation system in C4 plants (Yue, 1978) requires higher energy input, making photosynthesis more susceptible to impairment (Xu et al., 2016). Previous research (Liu, 2020) has shown that low-light stress significantly suppresses maize photosynthetic capacity by damaging photosystems and limiting carbon assimilation. In this study, after 15 days of symbiotic growth, the net photosynthetic rate of maize decreased due to shading by tobacco leaves. At this early stage, maize remained in the seedling phase and relied mainly on seed reserves (Gao, 2006; Kc et al., 2013); therefore, the impact of low-light stress was limited. As symbiosis progressed, nutrient demand increased, strengthening dependence on both soil nutrients and photosynthesis. By mid-symbiosis (25 days), the net photosynthetic rate in relay-cropped maize was lower than in monoculture, along with changes in internal photosynthetic regulation. PPDK and PEPC are key rate-limiting enzymes in C4 photosynthesis (Fang et al., 2024), whereas Rubisco is the main CO_2_-fixing enzyme in the C3 cycle. Previous studies (Fang et al., 2024; Sun et al., 2017; Zhang, 2020) report reduced activities of these enzymes under low light, consistent with our results. With prolonged symbiosis, the activities of PPDK, PEPC, and Rubisco in maize leaves were significantly lower than in monoculture plants. Rubisco, which catalyzes CO_2_ fixation in the C3 pathway (Cui et al., 2025), represents the primary entry point of inorganic carbon into the biosphere. At 25 days, its activity was markedly reduced in the symbiosis group, indicating strong inhibition of C3 carbon assimilation. PPDK, which regenerates the CO_2_ acceptor in the C_4_ cycle (Fang et al., 2024), showed reduced activity at 15 days, suggesting early suppression of the C_4_ pathway. In contrast, PEPC—the major carbon-fixing enzyme in the C4 pathway (Liao et al., 2024) declined significantly only at 35 days, indicating that inhibition of C4 carbon fixation remained limited until the later stage of symbiosis.

### 4.3 Effects of relay cropping with tobacco on the expression of key genes associated with in photosynthesis in maize at harvest stage

Most crops fix carbon via the C3 cycle, whereas maize operates both the NAD-ME C_4_ pathway and the C_3_ cycle (Bellasio and Griffiths, 2014), enabling more efficient photosynthesis. In the NAD-ME pathway, carbon fixation is largely regulated by PEPC and PPDK (Sun et al., 2020), which are common transgenic targets for improving C3 crop yields (Zhang et al., 2006). In our study, we identified two differentially expressed PEPC genes (*PEPC*) and two PPDK genes (*PPDK1*, *PPDK2*) in both monoculture and relay-cropped maize.The dark reactions depend on ATP generated during the light reactions (Caffarri et al., 2014; Galka et al., 2012; Su and Li, 2024), which supports CO_2_ production and the synthesis of carbon-fixing enzymes. Weak light disrupts thylakoid structure and reduces the activity of thylakoid-associated enzymes (He et al., 2018). Consistently, Syvertsen et al. showed that low light reduces leaf carboxylase levels and photosynthetic intermediates (Syvertsen and Smith, 1984). In our study, *PPDK* and *PPC* genes were downregulated in relay-cropped maize, with a corresponding decrease in *PEPC* activity. This aligns with Su (Su and Li, 2024), who reported that *PEPC* and *PPDK* expression is highly light-sensitive. At 25 days, *PPDK* activity was significantly lower in relay cropping than in monoculture, matching its gene expression pattern. Significant differences in *PPC* activity appeared only at 35 days, also consistent with gene expression. These results indicate that weak light inhibits both *PEP* synthesis (the primary CO_2_ acceptor) and the conversion of CO_2_ to oxaloacetate in relay-cropped maize, with a stronger effect on PEP synthesis, consistent with Li (Li et al., 2024). In the C3 pathway, Rubisco is essential for carbon assimilation (Zhou, 2024), but its activity declines under weak light (Kc et al., 2013) (Kc et al., 2013). Here, the expression patterns of the Rubisco enzyme gene (*rbcL)* and the PRK gene (*At1g32060*) mirrored those of PEPC and PPDK. These enzymes are central to carbon assimilation and the synthesis of primary CO_2_-fixing enzymes in the C3 cycle. Consistent with findings by Yuan et al (Liu et al., 2025) reported that weak light stress reduced the Rubisco, PEPC, and PPDK activities in relay cropping maize, we observed downregulation of these genes in relay cropping, accompanied by reduced enzyme activities. In addition, donor-side limitation of PSI under weak light likely suppresses these enzymes by restricting electron transport and ATP production. Overall, these results indicate that weak light stress in relay-cropped maize primarily limits carbon assimilation and initial CO_2_ fixation. Interestingly, in relay-cropped maize, several genes involved in both the C3 and C4 pathways—including *tktA, FBP, ALDO, GAPA, PGK*, and *meaB*—were upregulated at 25 and 35 days. These genes encode enzymes that participate in glycolysis (Zhao et al., 2022; Meng et al., 2022, 2022; Zhao et al., 2022), a metabolic pathway closely associated with plant responses to abiotic stress (Zhang and Yang, 2011). Under stress, plants often increase the expression of glycolytic genes to improve metabolic efficiency, mobilize stored nutrients, and maintain essential physiological functions (Meng, 2017). Glycolysis of fructose under stress requires multiple enzymes with catalytic and energy-regulatory roles (Li et al., 2024; Zhang and Yang, 2011). In this study, shading by tobacco leaves reduced light intensity in relay-cropped maize compared with monoculture, decreasing chemical energy conversion during the light reactions and consequently limiting ATP supply for the dark reactions of both the C3 and C4 cycles. To mitigate these constraints, plants modulate sugar-signaling pathways (Chen et al., 2022), consistent with our findings. Relay-cropped maize compensated for reduced light-driven ATP production by upregulating glycolysis-related genes, including *tktA, FBP, ALDO, GAPA, PGK*, and *meaB*. This response enhanced the conversion of monosaccharides into pyruvate and ATP, thereby supporting continued photosynthesis and physiological metabolism under low-light conditions.

## 5 Conclusions

This study compared maize grown under two planting systems: relay cropping with tobacco during its harvesting period and monoculture. The activities of key photosynthetic enzymes in maize, including Rubisco, PPDK, and PEPC, and photosynthetic parameters, including net photosynthetic rate, stomatal conductance, and transpiration rate, were consistently lower in relay-cropped maize than in monoculture. Transcriptome analysis identified 3200 DEGs. KEGG and GO enrichment analyses indicated that most DEGs were associated with photosynthesis. These results suggest that relay-cropped maize experiences weak-light stress under the tobacco canopy, which suppresses carbon assimilation and reduces the synthesis of primary CO_2_ fixation enzymes, including PEP (C4) and ribulose-1,5-bisphosphate (C3), as well as their initial CO_2_ fixation products oxaloacetate (C4) and PGA (C3). Further analysis of the C3 and C4 pathways showed upregulation of genes encoding enzymes involved in glycolysis. Maize relay-intercropped with flue-cured tobacco is shaded by the taller crop and enhances glycolytic metabolism to sustain physiological processes under reduced photosynthesis. These findings provide practical guidance for determining suitable co-growth periods and for understanding photosynthetic regulation in C4 plants during the symbiosis period. Photosynthetic index analyses of maize subjected to different tobacco–maize symbiosis durations (0–35 days) indicated that the suitable symbiosis period is 0–25 days. After 35 days of coexistence, grain yield differed significantly between monocropped and intercropped maize, indicating yield loss under prolonged coexistence. In contrast, most photosynthetic indices already differed significantly at 25 days, suggesting that this time point represents a critical threshold. Therefore, in tobacco relay-intercropped maize, the symbiotic duration between the two crops should be maintained within 0–25 days.

## Data Availability

The datasets presented in this study can be found in online repositories. The names of the repository/repositories and accession number(s) can be found in the article/**Supplementary Material**.

## Supplementary material

SUPPLEMENTARY TABLE 1 Read alignment statistics for the Treatment Group with the Maize Reference Genome.
