# Supplementary material for: Integrated physiological and molecular insights into photosynthetic responses of maize following relay-cropping of tobacco
Source: Front Plant Sci. 2026 May 7;17:1787851. doi: 10.3389/fpls.2026.1787851 (PMC13190397; doi:10.3389/fpls.2026.1787851)
Supplement: SUPPLEMENTARY TABLE 1 — Read alignment statistics for the Treatment Group with the Maize Reference Genome. [file Table1.docx]

| Gene | Sequence (5'->3') | Sequence (3'->5') |
| --- | --- | --- |
| Zm00001eb039150_T001 | AGCAGCTAATCGTGTGGCTT | CGGCTAGTTCAGCACTCCAT |
| Zm00001eb072030_T001 | AGAGAGACATGGGAGAGCGT | TGCTTCTGCGGCTCAATGTA |
| Zm00001eb287770_T001 | AAACGTACGGGCAAAAGTGC | CCGACGGGTTCTCAAACTGA |
| Zm00001eb333420_T001 | CGTGAACGACCAGTTCCTCA | TCATGTAGTGCGTGTAGCGG |
| Zm00001eb349810_T001 | GCGGCGATCAAGATGGTAGA | TCTCCGCCCTCACCAGAATA |
| Zm00001eb398370_T001 | TGTCGACGGATCACAACAGG | CGCATGGTATCTTGGCGTTG |

Table S1 Gene-specific primers used for qRT-PCR of differentially expressed genes
