# Supplementary material for: Integrated physiological and molecular insights into photosynthetic responses of maize following relay-cropping of tobacco
Source: Front Plant Sci. 2026 May 7;17:1787851. doi: 10.3389/fpls.2026.1787851 (PMC13190397; doi:10.3389/fpls.2026.1787851)
Supplement: Supplementary file 2 [file Table2.docx]

Table S2.Table of alignment of Reads in the Treatment Group with the Maize Reference Genome

| Sample | Total | Raw Datas | Clean Data(%) | Adapter(%) | AF_Q20(%) | AF_Q30(%) | Unique_Mapped(%) | Multiple_Mapped(%) |
| --- | --- | --- | --- | --- | --- | --- | --- | --- |
| IM15-1 | 40671370 | 41560712 | 41216554(99.17%) | 27520(0.07%) | 6007189017(98.30%) | 5810873429(95.09%) | 33625937 (82.68%) | 1651811 (4.06%) |
| IM15-2 | 40027982 | 40899892 | 40542884(99.13%) | 23542(0.06%) | 5909978956(98.28%) | 5715008941(95.03%) | 33194353 (82.93%) | 1621972 (4.05%) |
| IM15-3 | 49117066 | 50089274 | 49689488(99.20%) | 30104(0.06%) | 7266931247(98.61%) | 7068010681(95.91%) | 40552550 (82.56%) | 2103475 (4.28%) |
| IM25-1 | 43570852 | 44653736 | 44330450(99.28%) | 30790(0.07%) | 6512440021(98.84%) | 6353273249(96.42%) | 36995811 (84.91%) | 1803479 (4.14%) |
| IM25-2 | 48513708 | 49527052 | 49044728(99.03%) | 50826(0.10%) | 7137455207(98.19%) | 6891261068(94.80%) | 41024397 (84.56%) | 1960721 (4.04%) |
| IM25-3 | 52423558 | 53450890 | 52984600(99.13%) | 53064(0.10%) | 7683785646(98.48%) | 7457799535(95.58%) | 44297620 (84.50%) | 2254711 (4.30%) |
| IM35-1 | 48004210 | 48811590 | 48417334(99.19%) | 39938(0.08%) | 7081124026(98.61%) | 6889874176(95.95%) | 40764438 (84.92%) | 1686616 (3.51%) |
| IM35-2 | 43078300 | 43993184 | 43515160(98.91%) | 65324(0.15%) | 6327170844(98.40%) | 6132549900(95.37%) | 36320499 (84.31%) | 1549959 (3.60%) |
| IM35-3 | 41244300 | 41827344 | 41573828(99.39%) | 53976(0.13%) | 5993075871(96.49%) | 5645358610(90.89%) | 35149747 (85.22%) | 1433224 (3.47%) |
| MM15-1 | 51582124 | 55885542 | 55456990(99.23%) | 45432(0.08%) | 8076203654(98.64%) | 7866573466(96.08%) | 41420602 (80.30%) | 2208471 (4.28%) |
| MM15-2 | 39440806 | 40401820 | 40069680(99.18%) | 32070(0.08%) | 5838562010(98.46%) | 5664367379(95.53%) | 32271048 (81.82%) | 1628157 (4.13%) |
| MM15-3 | 36454114 | 37099940 | 36773920(99.12%) | 29808(0.08%) | 5364063768(98.45%) | 5201883435(95.47%) | 30166049 (82.75%) | 1518851 (4.17%) |
| MM25-1 | 38966598 | 39736910 | 39385318(99.12%) | 28502(0.07%) | 5742503240(98.31%) | 5555754519(95.11%) | 32888778 (84.40%) | 1548939 (3.98%) |
| MM25-2 | 37792046 | 38530572 | 38159732(99.04%) | 32910(0.09%) | 5543740535(98.21%) | 5353799518(94.84%) | 30981432 (81.98%) | 1486459 (3.93%) |
| MM25-3 | 38467028 | 39115990 | 38827506(99.26%) | 23024(0.06%) | 5675025144(98.58%) | 5515083617(95.80%) | 31055351 (80.73%) | 1534349 (3.99%) |
| MM35-1 | 57432844 | 58267112 | 57835598(99.26%) | 34754(0.06%) | 8485166311(98.83%) | 8287928644(96.53%) | 48931020 (85.20%) | 2305689 (4.01%) |
| MM35-2 | 43198676 | 44008876 | 43613702(99.10%) | 43058(0.10%) | 6347153143(98.27%) | 6136823626(95.01%) | 36521518 (84.54%) | 1692217 (3.92%) |
| MM35-3 | 47747720 | 48553930 | 48093846(99.05%) | 43844(0.09%) | 7005986157(98.45%) | 6795487105(95.49%) | 40525348 (84.87%) | 1887061 (3.95%) |
